# Supplementary figures and images for: MicrobiomeCensus estimates human population sizes from wastewater samples based on inter-individual variability in gut microbiomes
Source: PLoS Comput Biol. 2022 Sep 23;18(9):e1010472. doi: 10.1371/journal.pcbi.1010472 (PMC9534451; doi:10.1371/journal.pcbi.1010472)

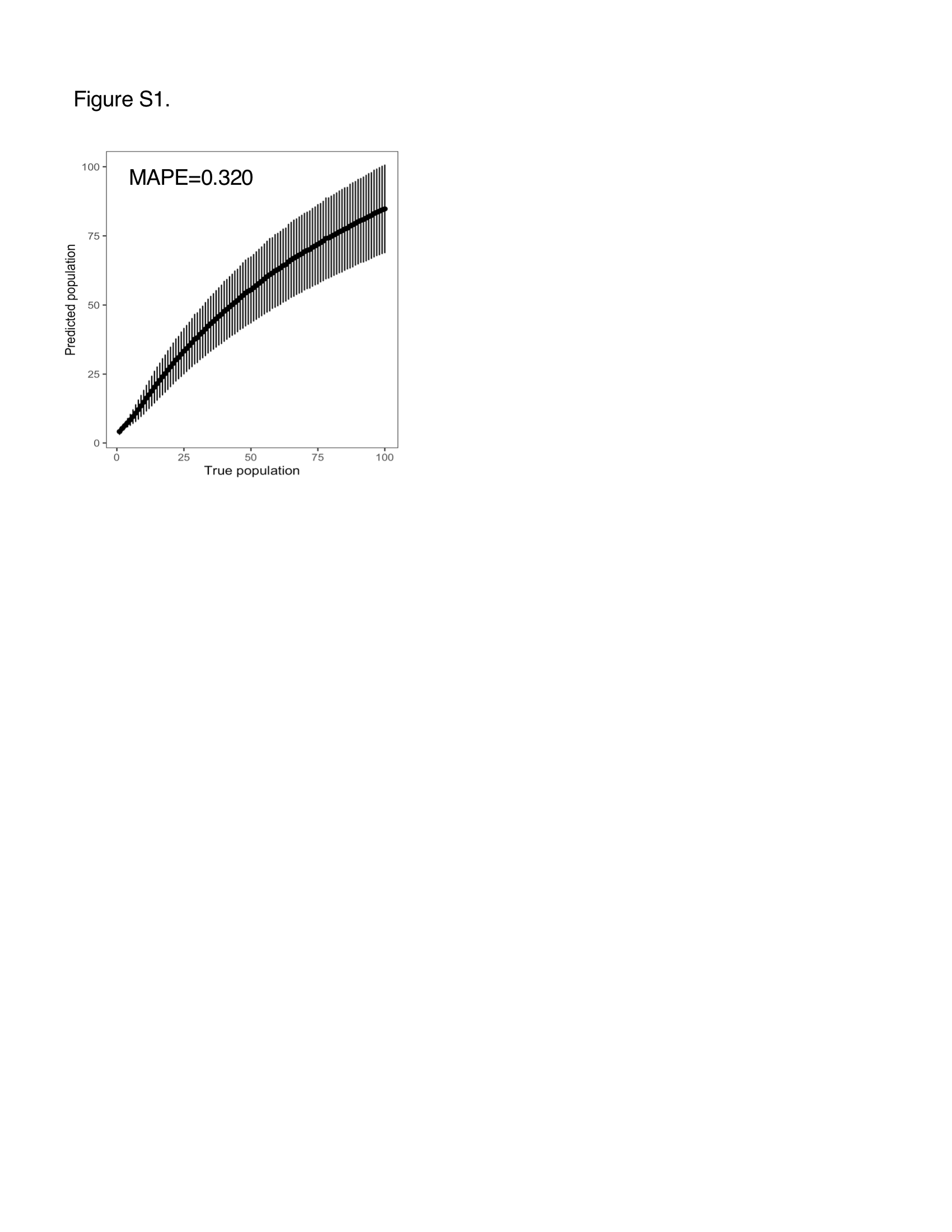

Supplement: S1 Fig — Black solid dots indicate the means of the predicted values, and error bars indicate the standard deviations of the predicted values. (TIFF) [file pcbi.1010472.s001.tiff]

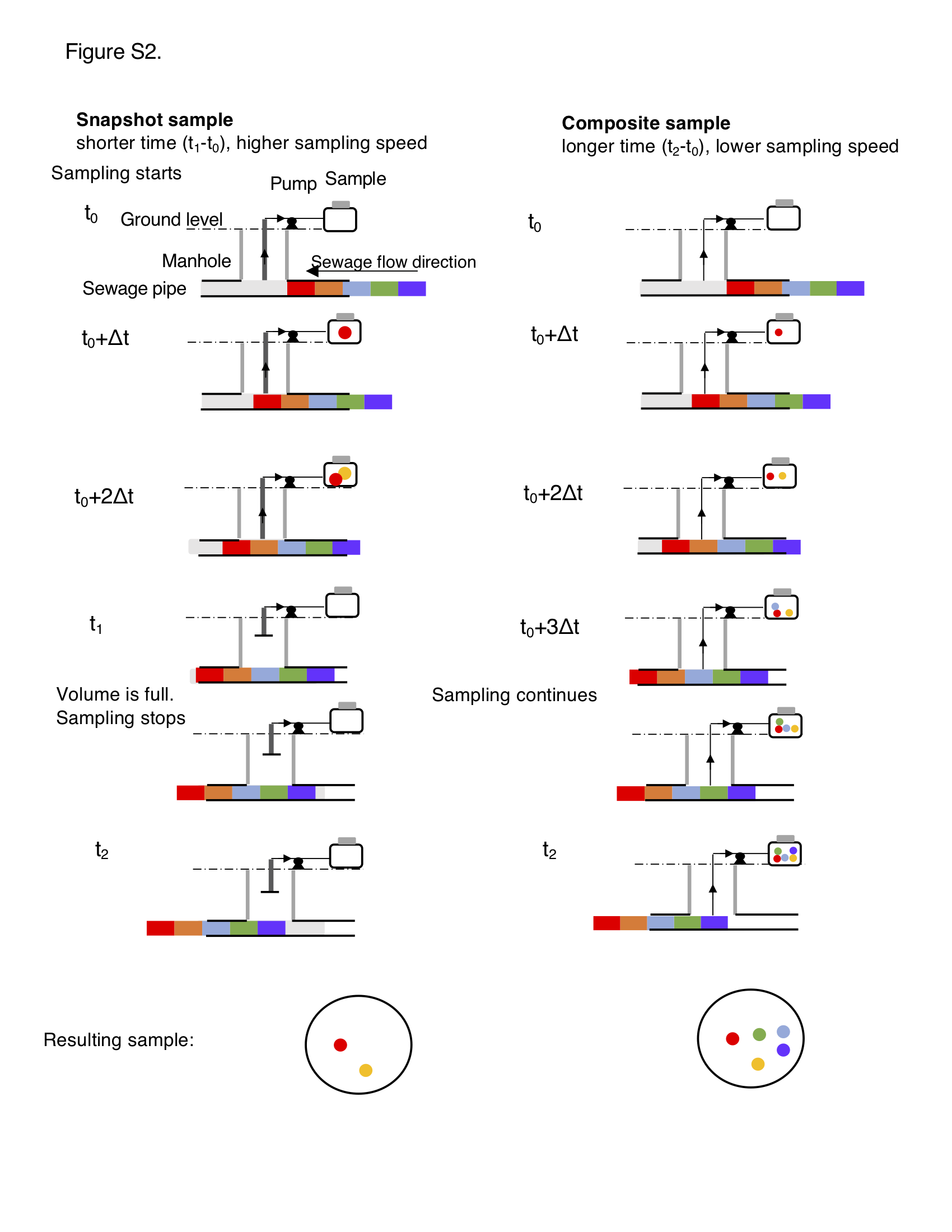

Supplement: S2 Fig — Sewage samples were collected from a manhole in upstream areas close to the sources. Snapshot samples were collected at a high pump speed for a short sampling time. Composite samples were collected at a low pump speed and for a long sampling time. Sewage flow is simplified to illustrate the sampling. Color bars indicate different sections of the flow, coming from different contributors. (TIFF) [file pcbi.1010472.s002.tiff]

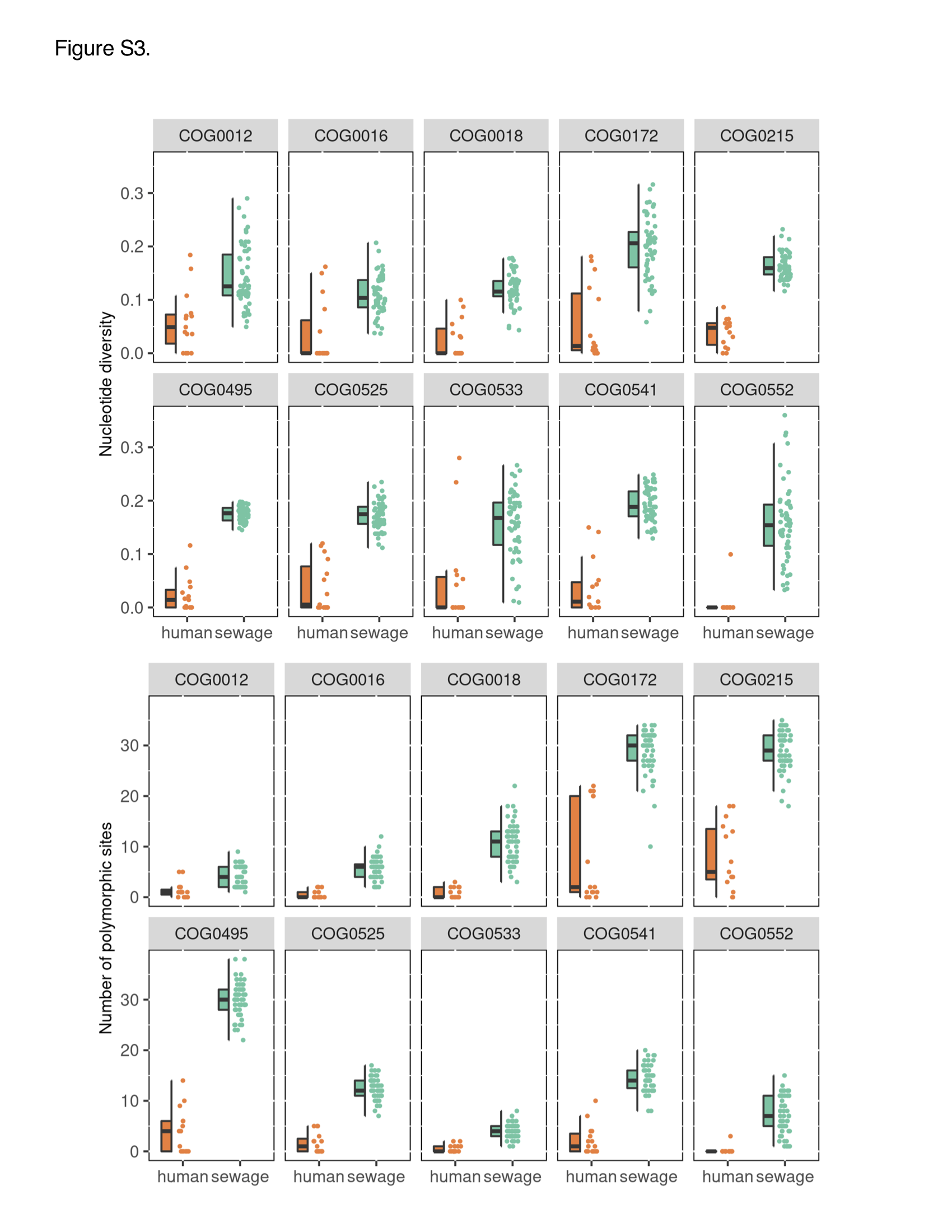

Supplement: S3 Fig — (TIFF) [file pcbi.1010472.s003.tiff]

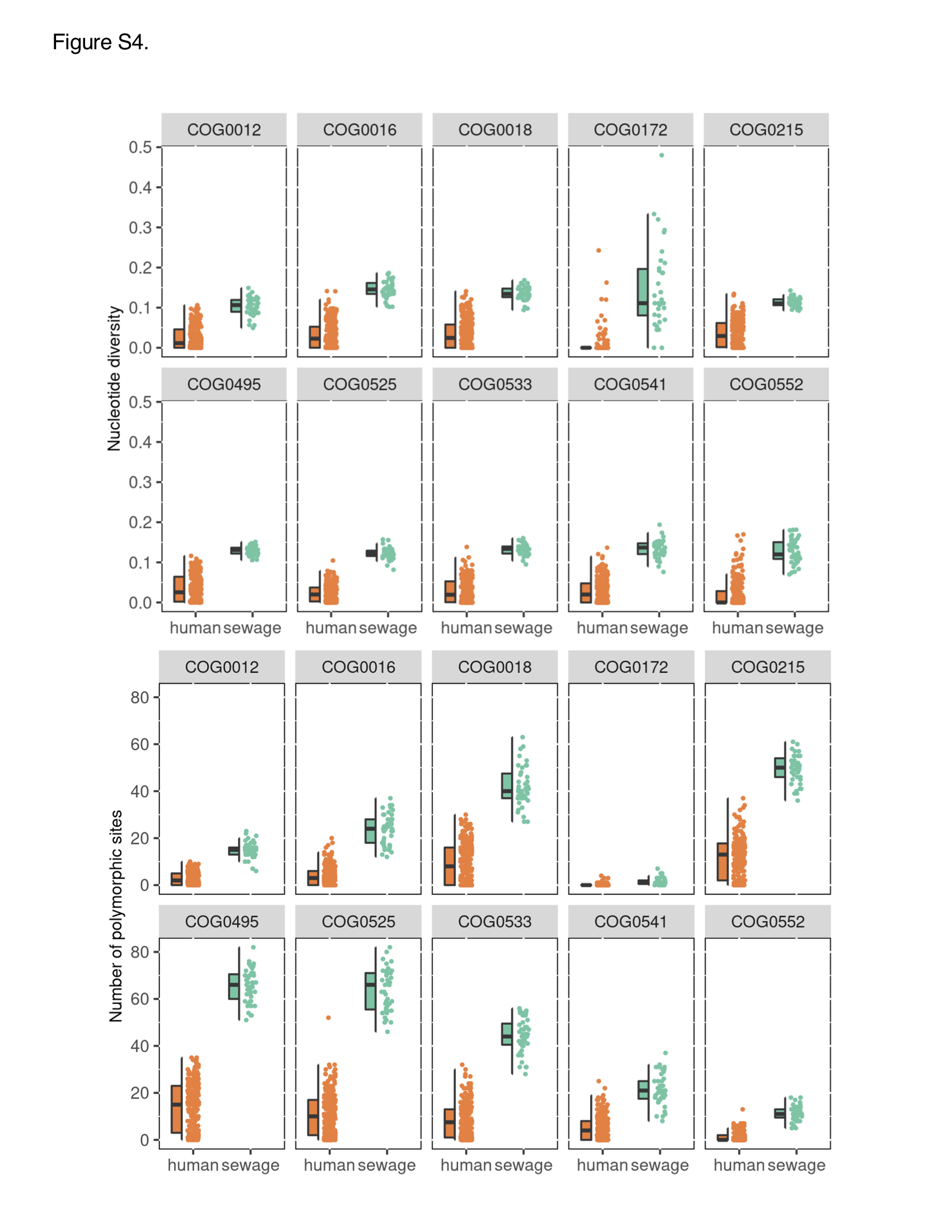

Supplement: S4 Fig — (TIFF) [file pcbi.1010472.s004.tiff]

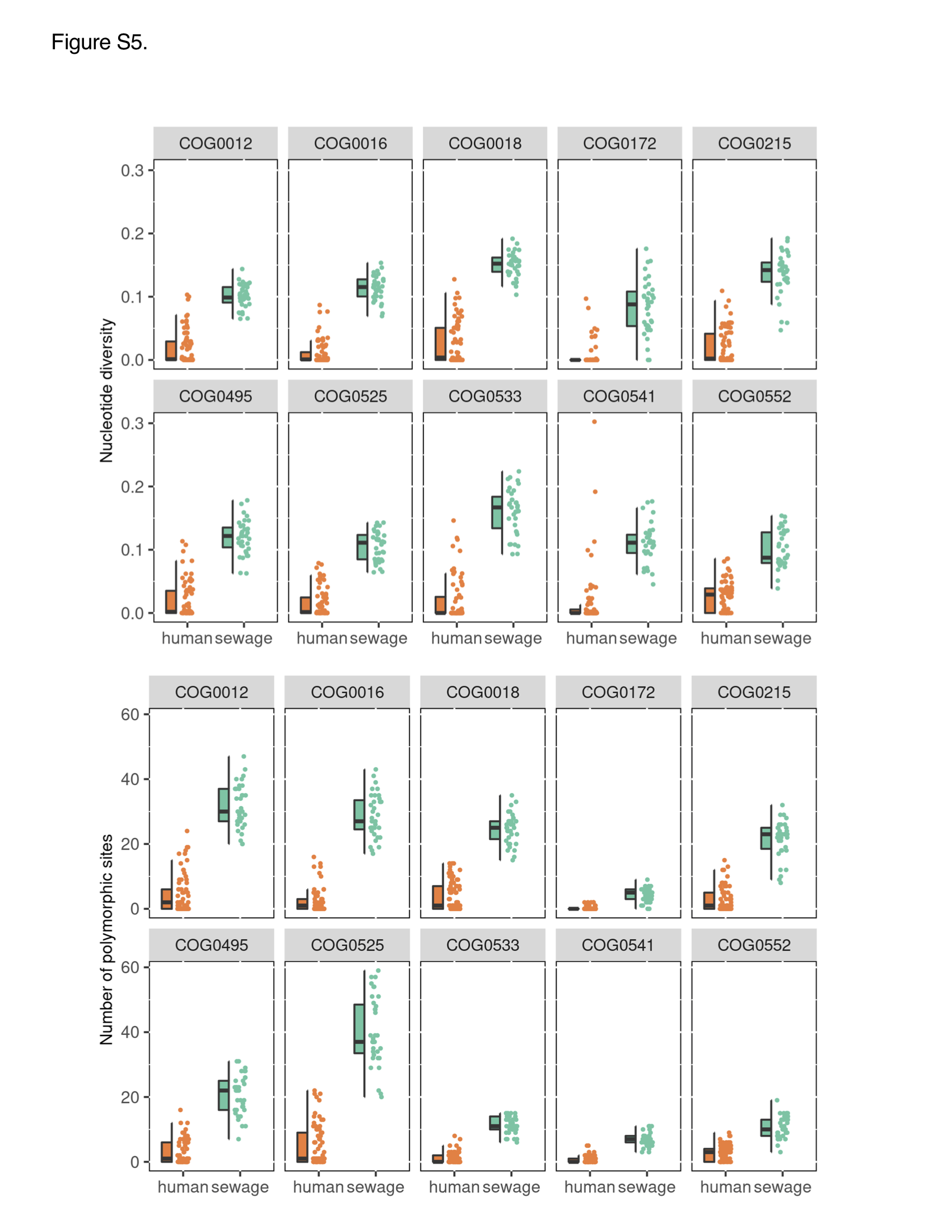

Supplement: S5 Fig — (TIFF) [file pcbi.1010472.s005.tiff]
