# Supplementary material for: MicrobiomeCensus estimates human population sizes from wastewater samples based on inter-individual variability in gut microbiomes
Source: PLoS Comput Biol. 2022 Sep 23;18(9):e1010472. doi: 10.1371/journal.pcbi.1010472 (PMC9534451; doi:10.1371/journal.pcbi.1010472)
Supplement: S1 Text — (DOCX) [file pcbi.1010472.s006.docx]

**S1 Text. Supplementary Texts**

1. **Supplementary Results**

**Sub-species diversity of gut-associated microorganisms in sewage and human gut microbiome samples**

To compare the sub-species diversity of gut-associated bacteria between human gut and sewage samples, we performed mapping and SNV calling using metagenomes collected from 1001 human subjects and 57 sewage samples against ten phylogenetic marker genes from representative genomes. Using the mOTUs2 pipeline, we filtered for SNVs that met a series of criteria and focused on species that were prevalent in both human gut and sewage microbiomes; this resulted in qualified SNV profiles from three gut-associated species, namely *Bacteroides vulgatus* (15 out of 1001 human samples and 55 out of 57 sewage samples), *Prevotella copri* (70 out of 1001 human samples and 35 out of 57 sewage samples), and *Eubacterium rectale* (246 out of 1001 human samples and 43 out of 57 sewage samples). For all three species above, sewage samples exhibited higher nucleotide diversity (Fig 4A-4C) and larger numbers of polymorphic sites than human gut samples (Fig 4D-4F). For instance, *Eubacterium rectale*, the most prevalent species in the dataset, showed a mean nucleotide diversity of 0.127 (s.d. 0.007) and 338 polymorphic sites (s.d. 38.21) in sewage samples, whereas a nucleotide diversity of 0.03 (s.d. 0.025) and 66 polymorphic sites (s.d. 48.82) in human gut samples (Fig 4). Higher nucleotide diversity and polymorphic sites in sewage were detected for each of the ten marker genes in these species when examined individually (Figs S3-S5).

To examine the effect of increasing population size on sub-species genetic variation in representative gut-associated microbial species, we simulated aggregate human gut samples using a sample without replacement procedure and computed the nucleotide diversity and numbers of polymorphic sites for the aggregate samples at different population sizes. This resulted in SNV profiles from 64 species. Our simulation showed increases in both nucleotide diversity and the number of polymorphic sites as more human gut samples were aggregated (Fig 4G and 4H). For instance, the nucleotide diversity and number of polymorphic sites in *Eubacterium rectale* increased from 0.029 (s.d. 0.026) to 0.149 (s.d. 0.002) and 64 (s.d. 54.33) to 1274 (s.d. 18.41), respectively, when the population size increased from 1 to 300. Further, the number of polymorphic sites strongly correlated with the population size (Pearson correlation coefficient > 0.8) in 49 out of the 64 species (S5 Table), suggesting the potential that the SNV profiles of a wide range of gut species could be developed into feature space for population size estimation. While the number of polymorphic sites showed a monotonic increase with population size, the increase in nucleotide diversity leveled off when the population size is greater than 50, suggesting that further mathematical developments are needed to harness the SNP data for population estimation.

1. **Supplementary Methods**

**Metagenomic sequencing and data analysis**

**B.1 Shotgun metagenome generation.**  Shotgun metagenomes of sewage samples were prepared using the same genomic DNA samples as the 16S rRNA sequencing experiments. The sequencing libraries were prepared using an Illumina Nextera Flex kit and sequenced using a Hiseq 2X150 format by the MIT BioMicro Center.

**B.2 Metagenome data quality control.** We acquired metagenomic sequencing reads from 1085 human gut samples (LifeLine Deep [[1]](https://www.zotero.org/google-docs/?BWGjNS)) and 76 sewage samples (this study), respectively. The sequencing reads in the human and sewage metagenome dataset ranged from 5,793,603 to 32,174,170 (mean=13,607,641, median= 12,839,746) and 81 to 24,483,901 (mean=9,358,345, median= 9,691,765), respectively. Metagenome samples were trimmed using Trimmomatic at a quality cutoff of 15, sliding window of 4 bp [[2]](https://www.zotero.org/google-docs/?g7KegN). Sequence duplicates due to amplification were removed using Fastuniq [[3]](https://www.zotero.org/google-docs/?pqQPFu). Upon quality filtering and deduplication, the metagenomes were subsampled to the same sequencing depth, 8,639,106 reads/sample, using Seqtk (https://github.com/lh3/seqtk) so that 90% of the samples from the entire dataset were retained. These quality control steps resulted in 1001 gut metagenomes and 57 sewage metagenomes qualified for further analyses.

**B.3 SNV mapping and calling.** Mapping was performed using the SNV workflow (map_snv) in the mOTUs2.5.0 pipeline against an alignment reference provided by the developers [[4]](https://www.zotero.org/google-docs/?x3CJHB). Briefly, the reference sequences were built from 87,991 reference genomes and 3,066 metagenomic samples grouped at the species level (mOTUs, species cutoff at 96.5% from 40 marker gene sequences, ref specI). To examine sub-species variation, ten universally occurring, protein-coding, single-copy phylogenetic marker genes (COG0012, COG0016, COG0018, COG0172, COG0215, COG0495, COG0525, COG0533, COG0541, COG0552) that had previously been validated for applications in metagenome studies were extracted. The alignment reference was then generated from the representative sequences from the species-level clusters (11,915 ref-mOTUs and 2,297 meta-mOTUs). Local alignment using BWA-MEM (v0.7.17) was performed with an alignment threshold set at 30. Matches shorter than 19bp were discarded.

Upon mapping, several criteria were set for SNV calling. First, low-quality bases, i.e., base quality lower than 15, were not considered. Next, an SNV was considered at a nucleotide position if (1) it had an allele frequency above 1%, which is considered as the classical definition of polymorphism, (2) and was supported by at least 5 reads (fc=5). These two criteria were set to filter out random sequencing errors. Third, additional filters were set on the percentage a reference is covered (i.e., breadth) as at least 10% per sample and per mOTU (fb=10) and the average coverage as at least 5 reads (fd=5). Last, an SNV was retained if 10% of the samples had at least 5 reads at the given nucleotide position (fp=0.1). This procedure resulted in qualified SNV profiles of 69 mOTUs and 13 mOTUs from the human gut dataset (S6 Table) and the sewage dataset (S7 Table), respectively.

**B.4 Comparison of inter-species diversity between sewage and human metagenomes.** SNV profiles from species that are present in at least three samples from both the sewage and human categories were compared for nucleotide diversity and the number of polymorphic sites.

**B.5 Simulation to illustrate sub-species diversity increase resulting from increasing population size.**  A random sampling without replacement procedure was performed from 1001 quality-filtered, sub-sampled human metagenomes. Let S_m_ denote the merged bam file from m samples. The sampling was done as follows:

Step 1: Generate S1 by randomly taking one bam file from a pool of 1001 samples;

Step 2: Generate S2 by randomly taking two bam files from the pool, and merging the two bam files using Samtools (v1.9) merge command.

Step 3: Repeat Step 2 for m = 3, 5, 10, 20, 50, 100, 200, 500.

Step 4: Repeat Step 1-3 100 times.

SNP profiles were generated from the resulting merged bam files following the same conditions as described above and used to compute nucleotide diversity and the number of polymorphic sites.

**C. References**

1. [Zhernakova A, Kurilshikov A, Bonder MJ, Tigchelaar EF, Schirmer M, Vatanen T, et al*.* Population-based metagenomics analysis reveals markers for gut microbiome composition and diversity. Science. 2016;352(6285):565–569. doi: 10.1126/science.aad3369.](https://www.zotero.org/google-docs/?9mAlZj)

2. [Bolger AM, Lohse M, Usadel B. Trimmomatic: a flexible trimmer for Illumina sequence data. Bioinformatics. 2014;30(15):2114–2120. doi: 10.1093/bioinformatics/btu170.](https://www.zotero.org/google-docs/?9mAlZj)

[3. Xu H, Luo X, Qian J, Pang X, Song J, Qian G, et al*.* FastUniq: A Fast De Novo Duplicates Removal Tool for Paired Short Reads. PLoS ONE. 2012;7(12). doi: 10.1371/journal.pone.0052249.](https://www.zotero.org/google-docs/?9mAlZj)

[4. Milanese A, Mende DR, Paoli L, Salazar G, Ruscheweyh HJ, Cuenca M, et al*.* Microbial abundance, activity and population genomic profiling with mOTUs2. Nat Commun. 2019;10(1):1014. doi: 10.1038/s41467-019-08844-4.](https://www.zotero.org/google-docs/?9mAlZj)
